# Use of REP- and ERIC-PCR to reveal genetic heterogeneity of *Vibrio cholerae *from edible ice in Jakarta, Indonesia

**DOI:** 10.1186/1757-4749-4-2

**Published:** 2012-03-15

**Authors:** Diana E Waturangi, Ignasius Joanito, Yogiara Yogi, Sabu Thomas

**Affiliations:** 1 Faculty of Biotechnology, Atma Jaya Catholic University of Indonesia, Jalan Jenderal Sudirman 51, Jakarta 12930, Indonesia; 2 Cholera and Environmental Biology Lab, Rajiv Gandhi Centre for Biotechnology (Dept. of Biotechnology, Govt. of India), Trivandrum-695 014, Kerala, India

**Keywords:** *Vibrio cholerae*, *ERIC-PCR*, *REP-PCR*, *Edible ice*

## Abstract

**Background:**

*Vibrio cholerae *is the causative organism of waterborne disease, cholera. *V. cholerae *has caused many epidemics and pandemics of cholera for many years. In this study, *V. cholerae *recovered from edible ice were investigated for their genetic diversity using Enterobacterial Repetitive Intergenic Consensus (ERIC) PCR and Repetitive Extragenic Palindromic (REP) PCR. Isolation was done using selective medium and the presumptive isolates were confirmed through biochemical and serological assays.

**Results:**

Seventy-five isolates of *V. cholerae *were recovered from ice samples collected from different locations of Jakarta. Specifically, 19 of them were identified as O1 serotype, 16 were Ogawa, 3 isolates were Inaba and the remaining isolates were non-O1. The fingerprinting profiles of *V.cholerae *isolated from ice samples were very diverse.

**Conclusion:**

This result showed that the ERIC sequence is more informative and discriminative than REP sequence for analysis of *V. cholerae *diversity.

## Background

Cholera is characterized by severe watery diarrhea caused by toxigenic *Vibrio cholerae*, which colonizes the small intestine and produces an enterotoxin, the cholera toxin (CT).

*V. cholerae *is classified on the basis of somatic antigens (O) into serovars or serogroups, and there are at least 200 known serogroup. Two serogroups, O1 and O139, have been associated with epidemic disease [1]. Serogroup O1 thought to include all the strains responsible for epidemic and endemic cholera; it has two major serotypes, Ogawa and Inaba. Each of those serotypes can be further divided into two biotypes, classical and El Tor, based on biochemical properties and susceptibility to bacteriophages. Serogroup O139 appears to be a hybrid of O1 strains and non-O1 strains. However, this organism does not produce O1 LPS and lacks at least some of the genetic material necessary for production of O1 antigen [2].

In Indonesia, a total of 17 episodes of epidemic diarrheal disease were investigated from 1993 to 1999 and were found to be caused by *V. cholerae *O1 [3]. According to WHO report [4], there was a sharp increase in the number of cholera cases. A total of 131943 cases, including 2272 deaths, reported from 52 countries. Overall, this represents a 30% increase compared with the number of cases reported in 2004.

In Indonesia edible are often used in street food and are consumed almost every day. Although it is so commonly consumed, it may not be prepared properly. We suspect that it may be a major important concern and we conducted a study on the genetic diversity of *V.cholerae *isolated from this possible source of contagion.

The initial analysis of Repetitive Extragenic Palindromic (REP) sequences in *E. coli *showed that this sequence was frequently present in complex clusters of several copies. These clusters are present and transcribed in about 25% of transcription units and may account for as much as 1% of the total genome. The REP element has been shown to be located between genes within an operon or at the end of an operon, and in operons distributed throughout the *E. coli *genome [5].

From the analysis of DNA sequence databases, Enterobacterial Repetitive Intergenic Consensus (ERIC) sequence is found to be approximately 126 bp in length. Like the REP sequence, the ERIC sequence repeat includes a conserved inverted repeat [6] and is located in non-coding transcribed regions of the chromosome, in either orientation with respect to transcription.

ERIC-PCR has been chosen for intraspecies profiling to several bacteria, for instance *Bacillus anthracis *and *Bacillus cereus *[7], *Enterobacter sakazakii *[8], *Lactobacillus *[9], *Listeria monocytogenes *[10], *Salmonella enteritidis *[11,12]. According to [7] ERIC-PCR typing can provide more discriminative DNA patterns of *Bacillus anthracis *and *Bacillus cereus *strains. Comparison of ITS profiling, REP- and ERIC-PCR of *Salmonella enteritidis *showed that ERIC-PCR can give high discriminatory index [12]. On the other hand, ERIC-PCR was able to show species specific band that pulsed field gel electropheresis could not show it [9]. Those comparisons has revealed that ERIC-PCR is a powerful techniques and informative for intraspecies profiling.

Thus, the objectives of this experiment were to obtain information on the genetic diversity of *V. cholerae *from edible ice samples used in street food using ERIC-PCR, and to compare the effectiveness of REP-PCR and ERIC for analysis genetic diversity of *V. cholerae*.

## Results

We recovered 109 isolates of *V. cholerae *from 40 ice samples throughout Jakarta (Table 1). The serological assay classified 24 isolates as O1 serotype, and these are further differentiated into 20 Ogawa and 4 Inaba isolates. Of these 109 isolates, however, only 75 isolates were subjected to ERIC and REP-PCR.

**Table 1:** Origin of samples and number of positive isolates

Regional	Location of Sampling	Number of presumptive Isolate	Number of Positive Isolate
East Jakarta	A	6	-
	B	6	-
	C	6	1
	D	24	2
	E	12	3
	F	12	-
	G	23	1
	H	6	-

North Jakarta	I	10	3
	J	24	-
	K	10	-
	L	10	-
	M	9	4
	N	19	1
	O	24	-
	P	12	-

West Jakarta	Q	34	13
	R	33	1
	S	27	3
	T	27	1
	U	24	2
	V	24	10
	W	24	5
	X	24	8

South Jakarta	Y	5	-
	Z	6	-
	AA	10	5
	AB	24	22
	AC	24	2
	AD	24	2
	AE	24	3
	AF	24	-

Central Jakarta	AG	10	-
	AH	9	-
	AI	11	6
	AJ	12	-
	AK	24	8
	AL	12	3
	AM	12	-
	AN	4	-

Total isolates		617	109

It was collected once for each sample

**Figure 1:**
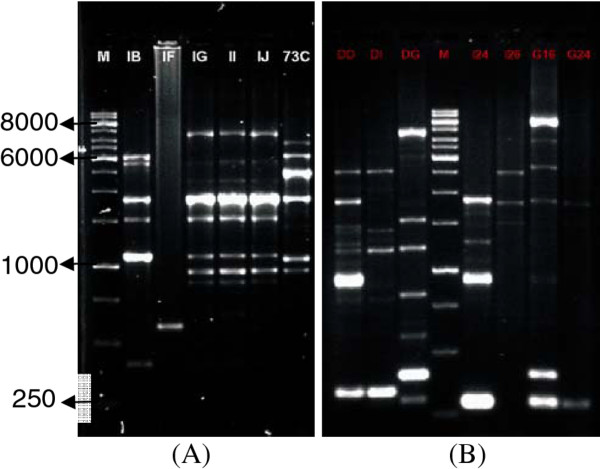
**DNA fingerprints of several environmental isolates of *V. cholerae *generated by ERIC PCR (A) and REP PCR (B) amplification**. M, marker 1 kb ladders (Fermentas).

The ERIC and REP PCR result of *V. cholerae *strains showed that the *V. cholerae *isolates were genetically diverse. For example, 31 different fragments of DNA ranging from 250 to 8000 base pairs were amplified with ERIC PCR (Figure 1A) and 33 different fragments ranging from 250 to 6000 base pairs were amplified with REP PCR (Figure 1B). As in a previous study by [13] the results obtained with ERIC PCR was less complex than REP PCR.

**Figure 2:**
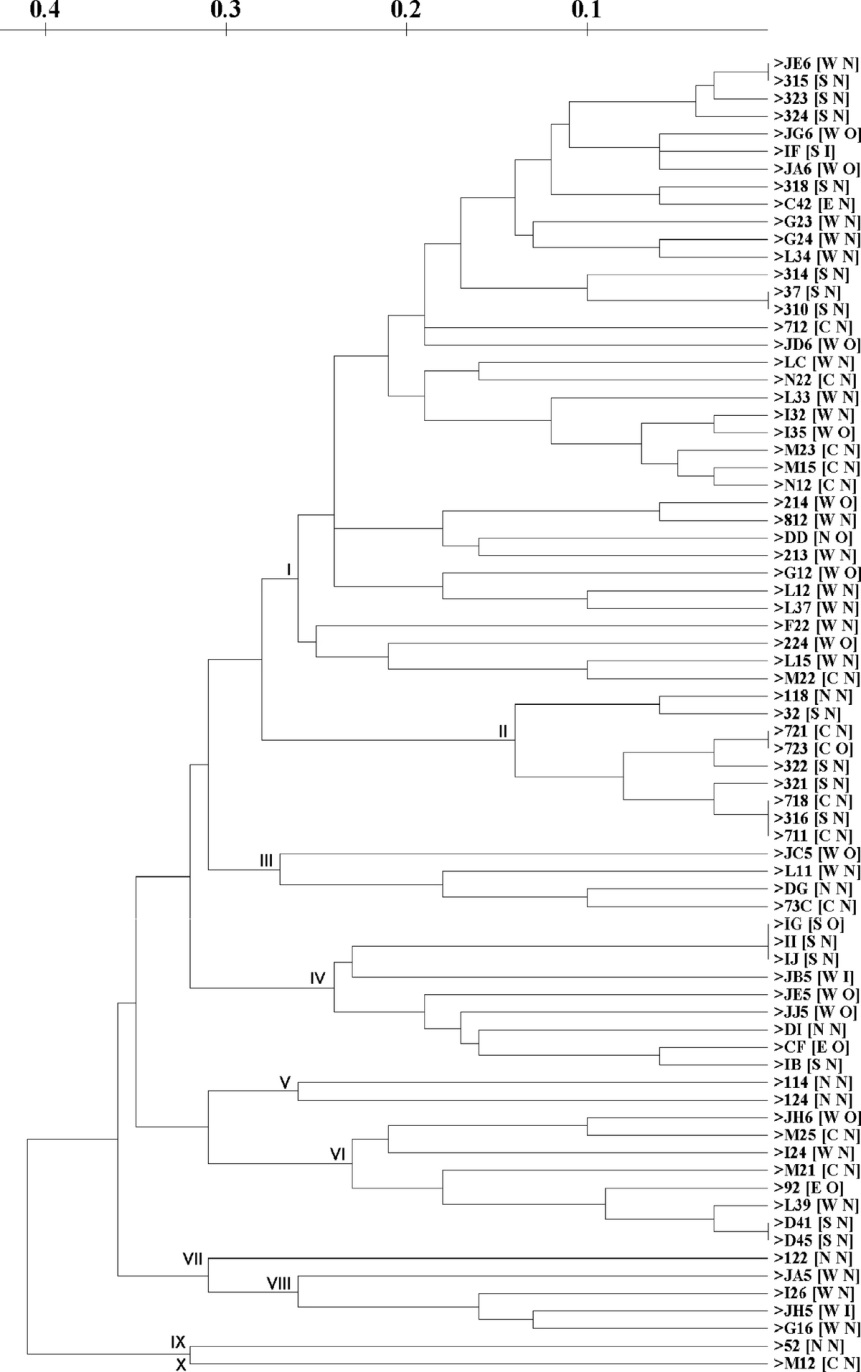
**Dendrogram of ERIC sequences using simple-match similarity matrix clustered by the unweighted pair-group method with arithmetic means**. W: west Jakarta, S: south Jakarta; N: north Jakarta; C: centre Jakarta; E: East Jakarta for first code in the bracket; O: Ogawa O1; I: Inaba O1; N: non-O1 for second code in the bracket.

The ERIC dendrogram result (Figure 2) showed that there are 10 different clusters with 0.72 simple match similarity. This result was obtained from 67 different DNA fingerprinting profiles out of 75 isolates. It indicated that there were some identical fingerprint profiles as shown in the isolate JE^6 ^and 315; isolate 37 and 310; isolate 721 and 723; isolate D41 and D45; isolate IG, II and IJ; and isolate 718, 711, and 316 (the origin of isolation and serotype information are shown in Table 2).

**Table 2:** Origin of sample and serotype of isolate that used in this study

Isolate	Regional	Location of Sampling	Serotype
CF	East Jakarta	C	Ogawa-O1
C42		D	Non-O1
92		G	Ogawa-O1

DD	North Jakarta	I	Ogawa-O1
DG			Non-O1
DI			Non-O1
114		M	Non-O1
118			Non-O1
122			Non-O1
124			Non-O1
52		N	Non-O1

F22	West Jakarta	U	Non-O1
G12		W	Ogawa-O1
G16			Non-O1
G23			Non-O1
G24			Non-O1
I24		X	Non-O1
I26			Non-O1
I32			Non-O1
I35			Ogawa-O1
L11		V	Non-O1
L12			Non-O1
L15			Non-O1
L33			Non-O1
L34			Non-O1
L37			Non-O1
L39			Non-O1
JA5		Q	Non-O1
JB5			Inaba-O1
JC5			Ogawa-O1
JE5			Ogawa-O1
JH5			Inaba-O1
JJ5			Ogawa-O1
JA6			Ogawa-O1
JD6			Ogawa-O1
JE6			Non-O1
JH6			Ogawa-O1
JG6			Ogawa-O1
LC		R	Non-O1
213		S	Non-O1
214			Ogawa-O1
224			Ogawa-O1
812		T	Non-O1

IB	South Jakarta	AA	Non-O1
IF			Inaba-O1
IG			Ogawa-O1
II			Non-O1
IJ			Non-O1
D41		AC	Non-O1
D45			Non-O1
32		AB	Non-O1
37			Non-O1
310			Non-O1
314			Non-O1
315			Non-O1
316			Non-O1
318			Non-O1
321			Non-O1
322			Non-O1
323			Non-O1
324			Non-O1

M12	Central Jakarta	AK	Non-O1
M15			Non-O1
M21			Non-O1
M22			Non-O1
M23			Non-O1
M25			Non-O1
N12		AL	Non-O1
N22			Non-O1
73C		AI	Non-O1
711			Non-O1
712			Non-O1
718			Non-O1
721			Non-O1
723			Ogawa-O1

There was no correlation between the origin of the isolates and the fingerprint profile. For example the fingerprint profile of isolate JE^6^, which was isolated from West Jakarta, is identical with that of isolate 315, which was isolated from South Jakarta (the distance between these two locations was approximate 10 km) and clustered in cluster I. However, isolate 118 and 122, which were isolated from the same place (M, North Jakarta) have very different fingerprint profiles and were in different clusters (118 in cluster II and 122 in cluster VII). Cluster I was very diverse, having isolates collected from all part of Jakarta. There was also no correlation between fingerprint profile and *V. cholerae *serotype. For instance, isolate 723 (Ogawa-O1 serotype) and isolate 721 (non-O1 serotype) had an identical fingerprint profiles and were in cluster II. However isolate JB^5 ^and JH^5 ^which have same serotype (Inaba-O1), have very different fingerprint profiles and clustered differently (JB^5 ^in cluster IV and JH^5 ^in cluster VIII).

**Figure 3:**
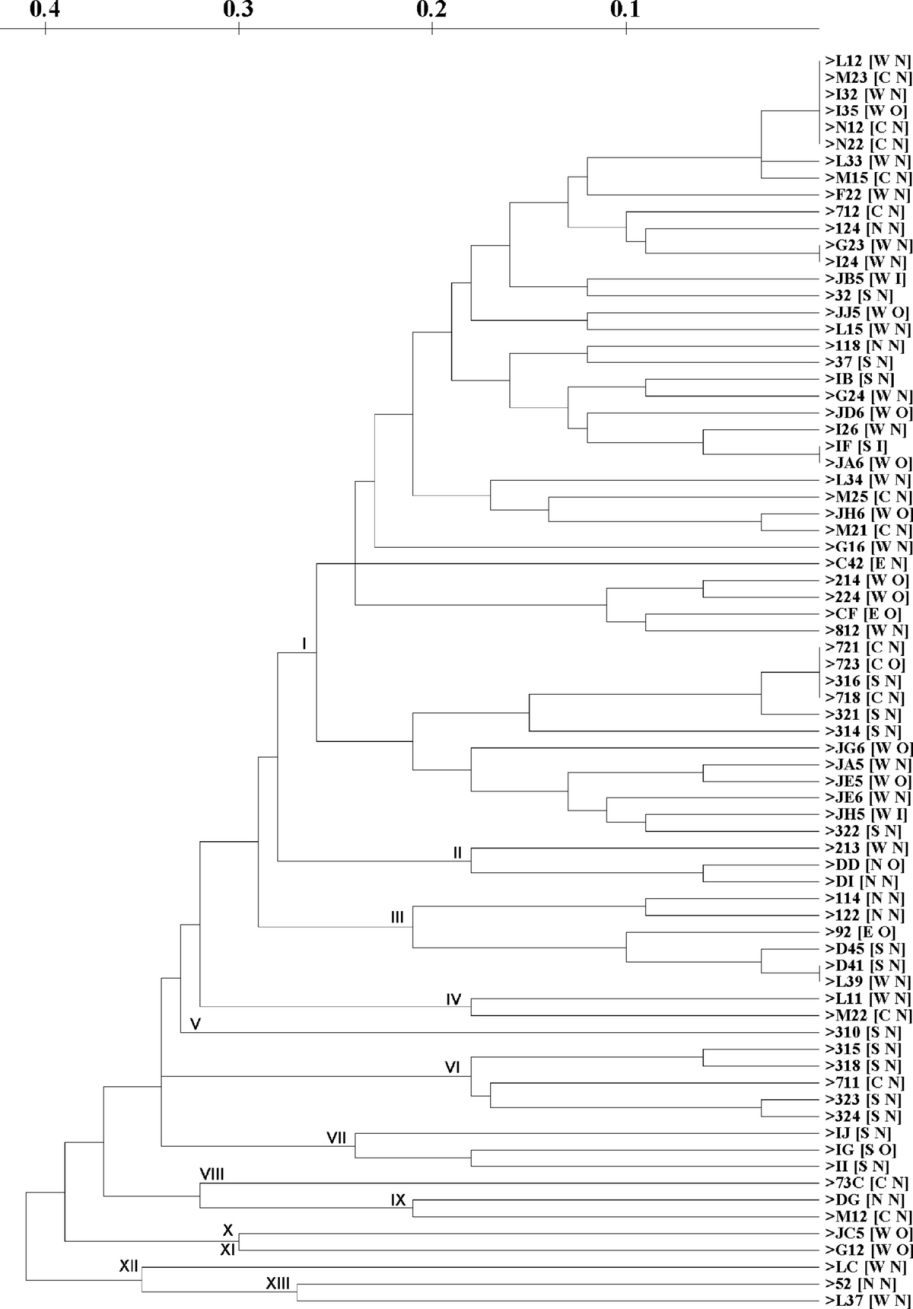
**Dendrogram of REP sequences using simple-match similarity matrix clustered by the unweighted pair-group method with arithmetic means**. W: west Jakarta, S: south Jakarta; N: north Jakarta; C: centre Jakarta; E: East Jakarta for first code in the bracket; O: Ogawa O1; I: Inaba O1; N: non-O1 for second code in the bracket.

Like the ERIC dendrogram, the REP dendrogram (Figure 3) also showed no correlation between the origin of isolation, *V. cholerae *serotype and fingerprint profile. But there were also some differences between them. The REP dendrogram formed more clusters, 13 with one major cluster (cluster I), compared to the ERIC dendrogram. But although there was an increase in cluster number, there was also a decrease in number of fingerprint profiles. This dendrogram only contained 64 different DNA fingerprint profiles. It indicated that, for REP, there were more isolates with identical fingerprint profiles, such as isolates L12, M23, I32, I35, N12, and N22; isolates G23 and I24; isolates IF and JA6; isolates 721, 723, 316, and 718; and isolates D41 and L39. It indicated that REP PCR cannot distinguish some isolate which can be differentiated with ERIC PCR.

**Figure 4:**
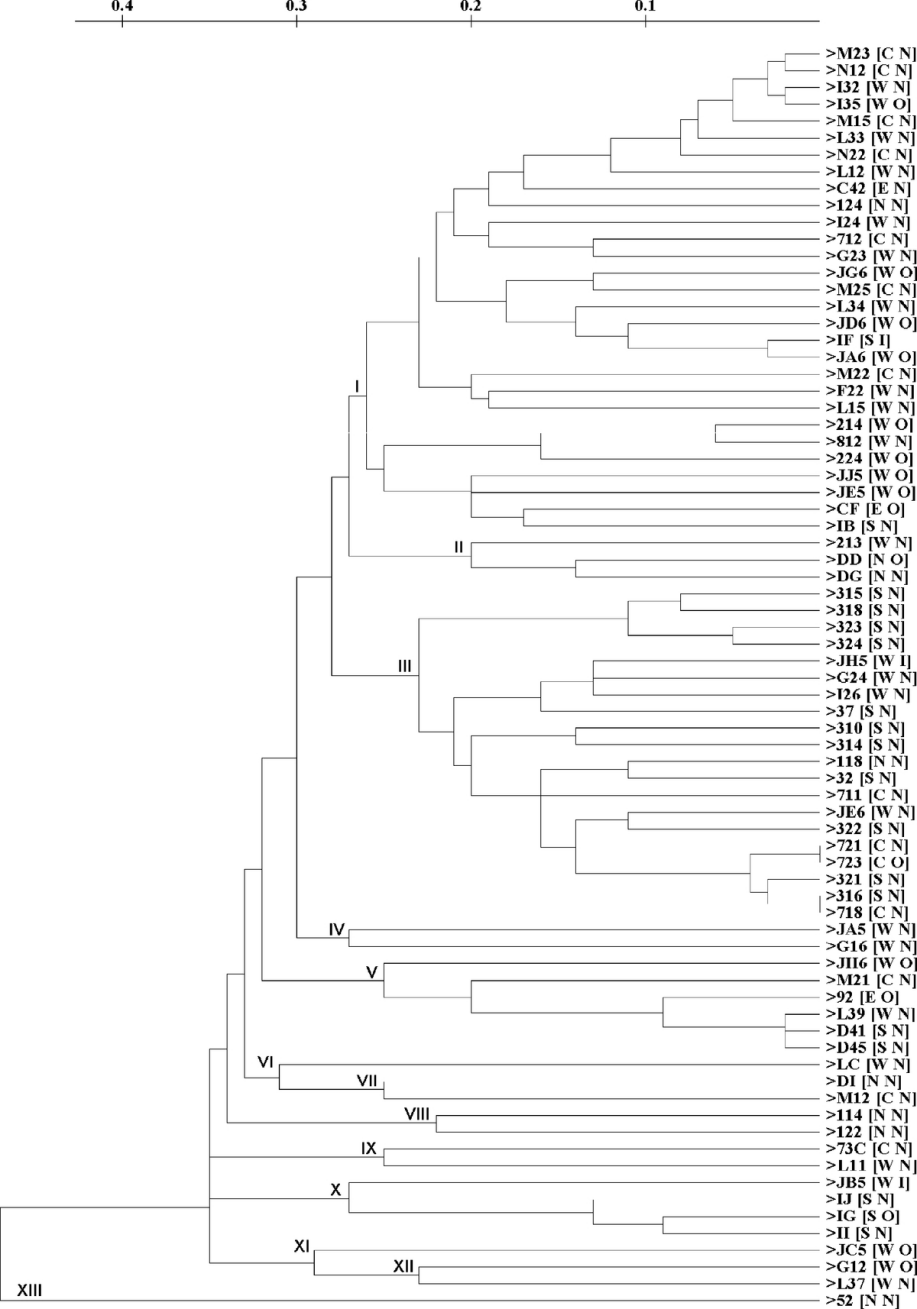
**Dendrogram of combined (ERIC and REP sequences) using simple-match 1 similarity matrix 2 clustered by the unweighted pair-group method with arithmetic means**. W: west Jakarta, S: 3 south Jakarta; N: north Jakarta; C: centre Jakarta; E: East Jakarta for first code in the 4 bracket; O: Ogawa O1; I: Inaba O1; N: non-O1 for second code in the bracket.

In this study, we also generated a dendrogram combining the ERIC and REP dendrograms (Figure 4). This dendrogram showed no correlation between origin of isolation, *V. cholerae *serotype and fingerprint profile. But there was an increase in the fingerprint profiles compared to either the ERIC or REP dendrogram (73 profiles). Only isolates 721 and 723; and isolates 316 and 718 have an identical profile. From this dendrogram we obtained 13 different clusters with two major clusters, cluster I and cluster III.

## Discussion

The results indicate that the hygiene level of edible ice in Jakarta is poor because it contains a large number of bacteria, including 109 isolates of *V. cholerae*. However, not all of ice samples were unhygienic because some samples only contained a few bacteria and no *V. cholerae *(Table 1). However, all samples were not unhygienic because some samples are devoid of *V. cholerae*.

Identical DNA profile can occur in different serotypes. One of the possible explanations for this is that the amplification site of ERIC-PCR and REP PCR are in non-coding sequences [13], while the somatic antigen upon which serotypes are based is encoded by the *rfb *operon [14]. Thus, it is suggested that the bacteria have similar non-coding sequences in that region or there were no ERIC or REP consensus sequences in that region, further research is needed.

Identical fingerprint profiles may also occur in samples of different origin, because the ice cubes in Jakarta may come from the same ice cube factory. Another possibility is that the water, which is used for making this ice, is collected from the same river. This result was similar to a previous study of *V. cholerae *[15] in that there was no correlation between genetic similarity and the geographical source of isolation.

The small chromosome of *V. cholerae *was quite conserved and stable outside of the superintegron region. In contrast to the general stability of the genome, the superintegron demonstrates pronounced divergence among toxigenic and nontoxigenic strains [16]. Thus we hypothesize that the clustering is based on another factors such as toxin production, antibiotic resistance or pathogenesis factor. Future research is needed to prove this hypothesis.

From this research we discovered that with REP sequences, the fingerprint patterns are more complex but the clustering result showed that the ERIC sequences are better, because the REP result shown only one big cluster and a small number of other cluster. The ERIC sequence is better than the REP sequence for analysis of *V. cholerae *samples, because it is less complex but more discriminative. This result is consistent with the comparison done by [12] in *Salmonella enteritidis*. A combination of ERIC and REP sequences can increase the level of discrimination for *V.cholerae*. This indicates that although there is an increase in discriminating ability, a combined dendrogram cannot provide better clustering than the ERIC dendrogram. In summary, many ice cube samples in Jakarta still contain *V. cholerae*. The fingerprinting profiles of *V.cholerae *isolated from ice cube in Jakarta were very diverse. There is also no correlation between the origin of samples the, bacterial serotype and the finger print profile. For an epidemiological study of *V cholerae*, the ERIC sequence is more discriminative than REP sequence.

## Materials and methods

### Samples collection

**Figure 5:**
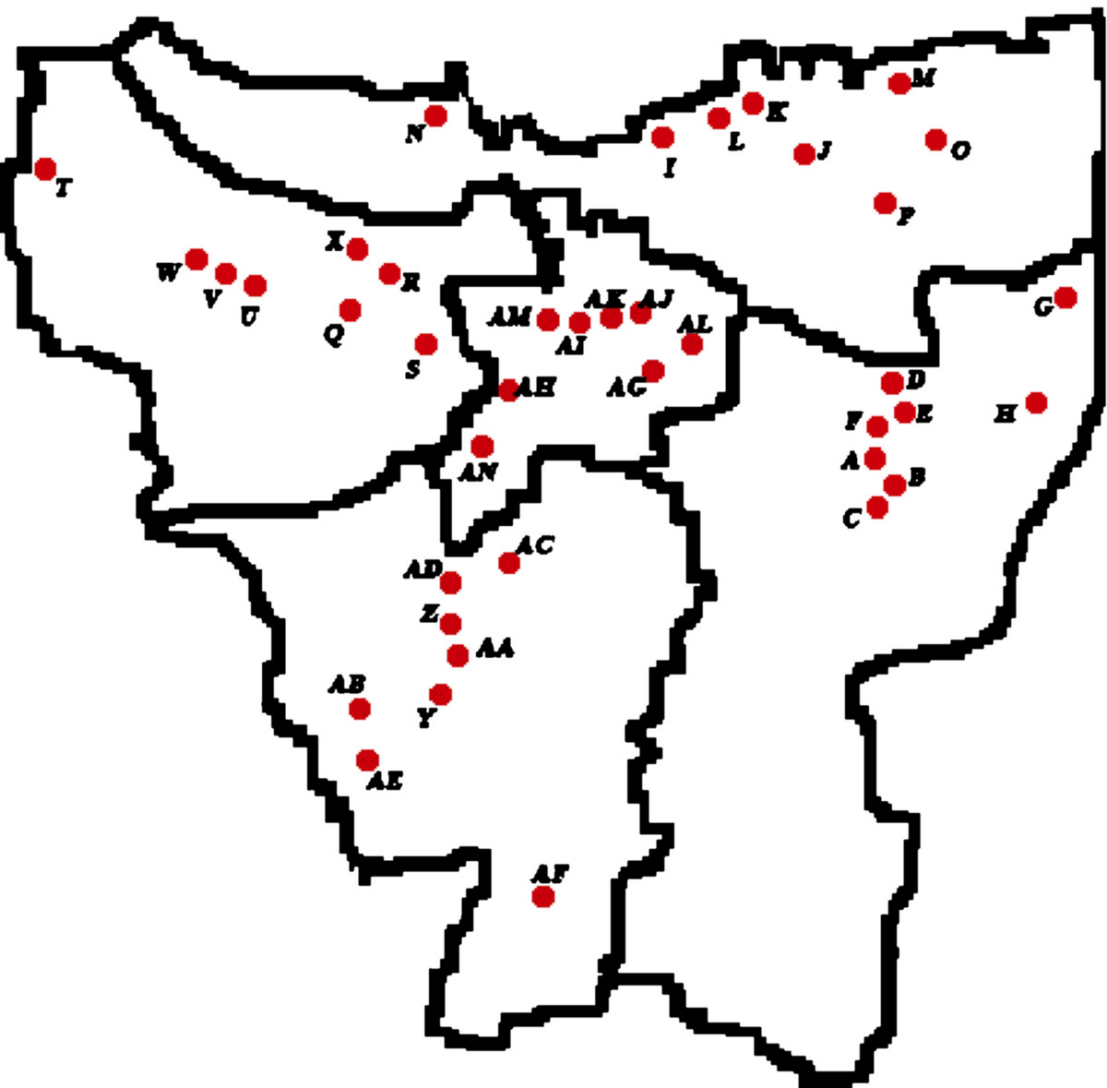
**Map of Jakarta showing the region which ice samples were collected**. For further information see also Appendices 1. (Scale 1:330000).

Ice samples were collected from ice suppliers around Jakarta (North, East, West, South, and Centre Jakarta) from October 2007 to May 2008. They were collected from eight ice suppliers from each part of Jakarta (Figure 5). The samples were put into cooler box and transported to the laboratory immediately.

### Isolation of *V. Cholerae*

Approximately 25 ml of melted ice was inoculated into 25 ml of *Alkaline Peptone Water *(APW) (Oxoid, England) and incubated at 37°C, 120 rpm, overnight. Then, 1 ml of inoculated APW was centrifuged at 1500 g for 3 minutes and the supernatant was removed; this step was repeated two times. The pellet was resuspended in 500 μl of 0.85% NaCl and subsequently diluted to 1:1000. Each of dilutions was spread onto Thiosulfate Citrate Bile-Salt Sucrose (TCBS) Agar (Oxoid, England).

### Biochemical and serological assays

About 5-8 suspected colonies were picked for further biochemical tests such as oxidase, Klinger Iron Agar (KIA) (Difco, USA) assay, Indole, and Lysine decarboxylase (Difco, USA) tests. The selected colonies were incubated at 37°C for 18-24 hours. To conduct the biochemical test, suspected colonies were grown on non-selective medium Brain Heart Infusion Agar (BHIA) (Oxoid, England). Serological assays were done by using *V. cholerae *polyvalent O antisera and monovalent Ogawa and Inaba antisera (Biofarma, Bandung Indonesia).

### Genetic diversity by using ERIC and REP-PCR based methods

The genomic DNA was extracted by standard methods [17]. PCR cocktails for 25 ml reaction mixtures contained 12.5 μl Go Taq (Promega, USA); 1 μl of 10 ρmol ERIC1R and ERIC2 [16]; or 1 μl of 10 ρmol REP 1R and REP 2I [18] (Table 3); 1 μl of DNA template; and 9.5 μl of double distilled H_2_O. The ERIC-PCR condition for this method followed [19] with some modification. For REP-PCR, the PCR followed the conditions [20]. The PCR result was visualized by running in a 1.8% agarose gel at 60 V for 2.5 hours.

**Table 3:** List of primers used in this study

No	Primer	Sequence	References
1	ERIC1R	(5'ATGTAAGCTCCTGGGGATTCAC 3')	[19]
2	ERIC2	(5'AAGTAAGTGACTGGGGTGAGCG 3')	
3	REP 1R	(5'-IIIICGICGICATCIGGC-3')	[18]
4	REP 2I	(5'-ICGICTTATCIGGCCTAC-3')	

## Competing interests

The authors declare that they have no competing interests.

## Authors' contributions

All authors read and approved the final manuscript.
